# Neutrophil CD64 as a diagnostic marker of sepsis in neonates: impact on clinical care

**DOI:** 10.1186/cc11764

**Published:** 2012-11-14

**Authors:** P Kingma, E Hall, D Marmer

**Affiliations:** 1 Cincinnati Children's Hospital Medical Center, The Perintal Institute, Cincinnati, OH, USA; 2 Cincinnati Children's Hospital Medical Center, Cancer and Blood Diseases Institute Laboratory, Cincinnati, OH, USA

## Background

Bacterial infections are a significant cause of morbidity and mortality in newborn infants. Successful treatment of neonatal infection depends on the early initiation of antibiotic therapy; however, unnecessary use of antibiotics increases bacterial resistance and has been associated with increased rates of necrotizing enterocolitis and death in premature infants. Unfortunately, the early clinical signs and symptoms of neonatal infection are often confused with other non-infectious conditions in premature infants such as apnea of prematurity and chronic lung disease. Neonatologists have traditionally relied on white blood cell counts (CBC) and bacterial cultures to help identify infected infants, but the CBC is an unreliable marker of infection in the neonatal population and bacterial cultures are too slow to be useful in the immediate evaluation of an infant.

## Methods

Therefore, in order to overcome these obstacles and improve the identification of infected infants, we incorporated neutrophil CD64 levels into the infection evaluations in our newborn ICU and evaluated the impact of this change on clinical care.

## Results

A total of 405 evaluations were performed in 268 infants (ages 1 to 293 days) from 2005 to 2009. Twenty-nine infants had culture-positive sepsis. The sensitivity and negative predictive value of the neutrophil CD64 assay was consistently ≥90% in identifying infected and non-infected infants. In fact, during the 3-year analysis period only three infants were identified with positive blood cultures that had a normal CD64 index. Two of these infants had blood cultures that were positive for *Staphylococcus epidermidis *and one had a positive blood culture that was obtained from a colonized central line. Although absolute CD64 levels did not correlate with severity of illness in our population (as determined by the need for ventilation (*P *= 0.87) or inotropic support (*P *= 0.90)), relative decreasing CD64 levels did correlate well with the resolution of infection within a given infant. When compared with infants with an elevated CD64 level, a normal CD64level decreased unnecessary antibiotic exposure by 3.9 days. By comparison, in infants evaluated with the traditional methods that did not include a CD64 level, a normal CBC only decreased antibiotic exposure by 1 day when compared with infants with an abnormal CBC.

## Conclusion

In summary, the clinical care that we provide our infants has improved with the use of neutrophil CD64 levels in our infectious evaluations.

